# A DNN-Based UVI Calculation Method Using Representative Color Information of Sun Object Images

**DOI:** 10.3390/s21227766

**Published:** 2021-11-22

**Authors:** Deog-Hyeon Ga, Seung-Taek Oh, Jae-Hyun Lim

**Affiliations:** 1 Department of Computer Science & Engineering, Kongju National University, Cheonan 31080, Korea; bigstring@smail.kongju.ac.kr; 2 Smart Natural Space Research Center, Kongju National University, Cheonan 31080, Korea; ost73@kongju.ac.kr; 3 Department of Urban Systems Engineering, Kongju National University, Cheonan 31080, Korea

**Keywords:** UVI, UV index, DNN, sky image, representative color, Mask R-CNN

## Abstract

As outdoor activities are necessary for maintaining our health, research interest in environmental conditions such as the weather, atmosphere, and ultraviolet (UV) radiation is increasing. In particular, UV radiation, which can benefit or harm the human body depending on the degree of exposure, is recognized as an essential environmental factor that needs to be identified. However, unlike the weather and atmospheric conditions, which can be identified to some extent by the naked eye, UV radiation corresponds to wavelength bands that humans cannot recognize; hence, the intensity of UV radiation cannot be measured. Recently, although devices and sensors that can measure UV radiation have been launched, it is very difficult for ordinary users to acquire ambient UV radiation information directly because of the cost and inconvenience caused by operating separate devices. Herein, a deep neural network (DNN)-based ultraviolet index (UVI) calculation method is proposed using representative color information of sun object images. First, Mask-region-based convolutional neural networks (R-CNN) are applied to sky images to extract sun object regions and then detect the representative color of the sun object regions. Then, a deep learning model is constructed to calculate the UVI by inputting RGB color values, which are representative colors detected later along with the altitude angle and azimuth of the sun at that time. After selecting each day of spring and autumn, the performance of the proposed method was tested, and it was confirmed that accurate UVI could be calculated within a range of mean absolute error of 0.3.

## 1. Introduction

As the time spent by modern people indoors is increasing, performing appropriate outdoor activities and receiving the necessary exposure to sunlight are necessary to maintain our health [1]. Consequently, information on the outdoor environment has become essential for each individual as environmental conditions can directly affect human health during outdoor activities. Major outdoor environmental conditions include the weather, atmospheric conditions, and fine dust. Recently, research interest in ultraviolet (UV) radiation, which has beneficial or harmful effects on the human body depending on the degree of exposure, has been growing [2,3]. UV radiation is electromagnetic radiation energy with a natural light wavelength ranging between 100 and 400 nm [4]. When the human body is excessively exposed to UV radiation, it causes skin diseases such as erythema and dermatitis; however, appropriate exposure has beneficial effects such as synthetic support of vitamin D and prevention of osteoporosis [5]. UV radiation is thus an important environmental factor for improving human health, and information on the UV radiation in our surroundings is recognized as necessary information to verify healthy outdoor activities [6]. Typically, people can grasp surrounding outdoor environment information such as wind, cloud, and air quality by simply watching the sky or the scenery of the outdoor terrain through their eyes. Detailed environmental information can be found through broadcasting, Internet, and API (Application Programming Interface)-based information services provided by governments or related agencies. However, unlike general environmental factors, UV radiation has wavelength properties of the invisible light band; hence, it is impossible to identify with the naked eye [7]. Furthermore, UV radiation information services of related agencies provide results collected through professional measurement equipment at the base measurement station, which does not provide UV radiation information at close locations for each individual [8]. Recently, several UV radiation-measuring instruments have been released to support the acquisition of more detailed UV radiation information from individual locations [9]. In addition, small sensors for UV radiation measurement or wearable devices equipped with a UV radiation sensor that operates in conjunction with smartphones have been released [10,11]. However, ordinary users find it difficult to use UV radiation measurements or devices because of the inconvenience of the purchase cost and that the operation of separate devices is necessary. Recently, there have been increasing attempts to derive environmental information such as weather and atmospheric conditions by analyzing after measuring the environment-related elements, which can be collected around users through small optical and image sensors that are easy to carry and use [12]. Shay Sosko collected environmental elements through optical, temperature, and humidity sensors embedded in mobile devices of residents in each region and then built regional weather maps based on the location values of GPS sensors [13]. Damien P. Igoe evaluated the total ozone column (TOC) by obtaining images and calibrating each pixel value after applying a narrow bandpass filter on a smartphone camera [14]. Marquez predicted the solar radiation of the Earth’s surface for a day through the analysis of images after shooting sky images [15]. Xiaoyang Liu proposed a method to estimate the concentration of fine dust in the area using pictures of the city center taken by a smartphone [16]. Recently, artificial intelligence and machine learning have been utilized in various engineering fields to solve problems in the living environment [17,18]. In addition, many cases of building deep learning models have been introduced which recognize and calculate useful environmental information by analyzing environment-related data collected through sensing technology. Afan Galih Salman predicted rainfall by inputting the wind, SOI (Southern Oscillation Index), SST (Sea Surface Temperature), and OLR (Outgoing Longwave Radiation) of ENSO (El Niño/Southern Oscillation) indicators through deep learning techniques [19], and Yubo Tao built a deep learning model that could predict the wind power of the next 48 h from the wind data of the previous three months [20]. Young-Soo Jo built a far-UV all-sky map by applying deep learning technology based on collecting the data from an extreme-UV image spectrometer mounted on a microsatellite (SATellite-1) [21]. Although several studies using optical or image sensors and deep learning have been conducted, few cases have applied related technologies to calculate the intensity of UV radiation, which is essential environmental information for human health.

Herein, a DNN-based ultraviolet index (UVI) calculation method is proposed using the representative color information of sun object images to provide UV radiation information at the user’s location. The proposed method first extracts the sun object regions by applying Mask R-CNN after taking a picture of sky images and extracts representative color information from the sun object images. Then, the DNN model is constructed to calculate the UVI by inputting the RGB color value, which is the representative color of the sun object images, and the location information of the sun. In addition, the proposed method is applied by selecting one of the days in spring and autumn, and performance evaluation experiments are conducted to compare the calculated results with the UVI measured through a spectroradiometer. This study introduces a new concept of the UVI calculation method that links and applies sky images, which are relatively easy to collect, and deep learning technology. It also provides a method to gather more accurate UV information at the user’s location by addressing the inaccuracy of UVI figures measured at a distance (based on the measuring station for each region).

## 2. UVI Calculation Based on Representative Color Information of Sun Object Images

**Figure 1:**
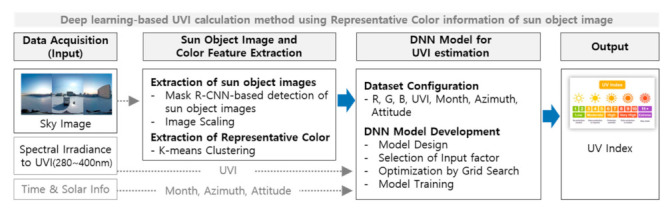
Process of the proposed method.

UVI is an index that indicates the intensity of UV radiation and is determined by the intensity of sunlight reaching the Earth’s surface. In addition, the intensity of sunlight reaching the Earth’s surface is affected by weather and atmospheric conditions, and weather and atmospheric conditions can be seen through sky images shot by a camera. Thus, the intensity of UV radiation can be extracted by analyzing images of the sky that continuously change according to season, weather, and time. Figure 1 shows the process of the proposed method.

As shown in Figure 1, the DNN model, which employed sky images as the main input, was constructed to calculate the UVI at the user’s location. The proposed method consists of three main processing steps. First, in the data acquisition step, sky images and UVI values were collected cumulatively under the same location and time conditions. Then, sun object images were extracted from inputted sky images followed by a process of detecting the representative color information of the sun object images through clustering of each pixel. Finally, the deep learning model, where the RGB color values of representative colors of the sun object images and the sun’s location and time information were used as the input, was constructed to calculate the UVI at the user’s location.

### 2.1. Collection of Sky Images and UVI Data

To extract the intensity information of UV radiation using sky images, sky images acquired at the same location at the same time and the measurement values of UVIs are required. A camera and a spectroradiometer (CAS 140 CT, Instrument Systems, Munich, Germany) were installed on the rooftop of the university building located at latitude 36.8522 and longitude 127.1509. An omnidirectional camera (Gear 360, Samsung, Seoul, South Korea, and VIRB360, Garmin, Olathe, KS, USA), which can acquire omnidirectional image data, was used to capture the position of the sun changing over time from sunrise to sunset. Here, the omnidirectional camera was fixed to look at the south-facing direction to measure the sky image without any change in direction or angle. A spectroradiometer is operated in connection with the solar tracking facility, considering that the solar phase and the intensity of UV radiation continue to change over time. The solar tracking facility was set to move according to the altitude and azimuth angles of the sun, and the optical receiver of the spectroradiometer was fixed to the tracking facility. Then, the sky images and spectral characteristics of the solar radiation at the same time were photographed and measured every minute. The data collection was conducted for a year from October 2019 to October 2020 and a total of 98,000 data points were obtained. The omnidirectional camera had wide-angle lenses on both sides, so two images (front and rear) were photographed at once. Because of this, the acquired two images were converted into one panoramic image, which allowed the viewing of image data from all directions. In addition, the UVI was calculated by applying Equation (1) to spectral power distribution (SPD) collected by a spectroradiometer.

(1) $$\mathrm{UVI}=k\int_{280}^{400}E\left(\lambda \right)\cdot W\left(\lambda \right)\;d\lambda ,(k=40)\;$$

Because the UVI is the intensity of UV radiation on the Earth’s surface considering the effect on human skin, the UVI was calculated through the integral after multiplying the erythemal weight by the spectral irradiance in the UV wavelength band (280–400 nm) as shown in Equation (1) [22]. Table 1 presents the hourly sky images and UVI for a specific day (5 April 2020) collected through measurement environments.

Table 1 verifies that sky images by time, especially the color and position around sun objects, changed from time to time. In addition, the UVI was relatively low at sunrise and sunset, whereas it was high at noon, showing a pattern of changing over time. This fact implied that the UVI could be calculated using color information of sun object images and position information of the sun. The UVI is heavily influenced by the intensity of sunlight reaching the Earth’s surface, so using the color information in the sun object region rather than using the background color of the entire sky image was advantageous for calculating the accurate UVI.

### 2.2. Extraction of Sun Object Images and Representative Colors

For accurate UVI calculation, the sun object region was detected and extracted without using the whole sky images, and then applied to the proposed method. Recently, many convolutional neural network (CNN)-based methods have been applied to object detection that extract the position and size information of specific objects such as people and faces within images [23]. In particular, a family of region-based convolutional neural network (R-CNN) models, which detect objects after generating a region of interest in images, have excellent performance [24], and Mask R-CNN can mask detected objects for each pixel, and it has the advantage of extracting exact positions and sizes because coordinate information is not lost when detecting objects [25]. 

**Figure 2:**
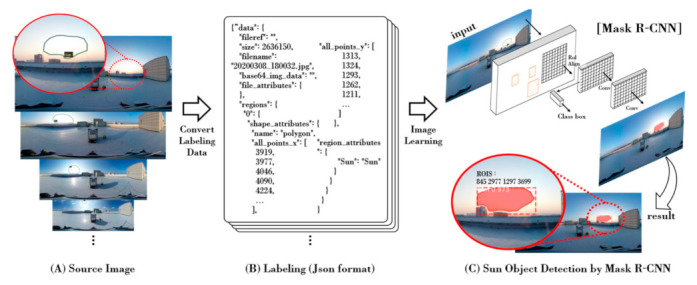
Process Mask R-CNN-based sun object detection process.

In this study, the Mask R-CNN technique was applied to extract accurate position and size information of sun objects in sky images. The Mask R-CNN model for detecting sun objects was implemented through Keras and Tensorflow in Python environments and a training process was conducted with training datasets with labeled data [26]. Generally, machine learning models related to object detection use COCO (Common Objects in Context) datasets as training datasets [27,28]. However, existing COCO datasets did not have labeled data of the sun in sky photos, which cannot be applied to the proposed method. In the proposed method, the labeled data of the sun was generated in the JavaScript Object Notation (JSON) format, which was added to the existing dataset, to detect sun objects. A total of 200 sky images were selected to label the sun considering various weather conditions and time. In the case of clear days, cloudless days were selected to extract sky images at various times from sunrise to sunset. In addition, in the case of cloudy days, various moments of the sun hidden by clouds were selected, and sky images of the respective 100 images for clear and cloudy days were selected, and labeling the sun was performed for the selected images. At this time, VGG Image Annotator (VIA) software was used to make it easy to annotate images, audio, and video sources [29]. When labeling, given the fact that many patterns of the sun covered by clouds or topography were observed, the sun boundary was drawn in the form of polygons rather than circles. Figure 2 is the process of detecting sun objects by applying the Mask R-CNN technique.

Figure 2A shows the sun in the sky images, and Figure 2B shows the result of labeling, which is converted to the JSON form. Figure 2C shows the result of detecting the sun object, which is a form similar to a pre-labeled polygon by inputting the sky image into the Mask R-CNN model. The part marked by the red mask is the sun object region, and the square dotted lines surrounding the region provide the position and size information of the sun object box. For the sun object image, a region for which horizontal and vertical lengths were twice those of the sun object box was extracted. Here, the sizes of the extracted sun object images were irregular. In the proposed method, image scaling was performed to normalize the sun object images to an equal size of 100 × 100 pixels for reliable processing when detecting the main color component (RGB) of the sun object image.

Deep learning technology is widely used in the field of detecting or classifying objects for images [30,31]. Deep learning technology can expect excellent performance if the same pattern is repeated regardless of position within images or the local characteristics of the proximity pixel region are distinct [32]. However, because few characteristic elements represent the intensity of UV radiation in sky images acquired through a general camera that captures the visible light range, it is very difficult to calculate the UVI by applying the existing image-based deep learning technology. However, as people can generally infer the burning degree of the sun or approximate weather after observing the sky before leaving their house, the feature information of the object image containing the sun can contribute to calculating the intensity information of UV radiation. The representative color was selected as a feature of pre-processed sun object images.

The representative color was determined by the color value of RGB (Red, Green, Blue) which showed the highest proportions in a 100 × 100-pixel sun object image, and it was applied as the main input element of the DNN model for calculating the UVI. The representative color extraction was implemented in python environments in the same way as in the preprocessing process, and the OpenCV2 library was used for initially loading images. Note that because the OpenCV2 library loads an array of images in the BGR order, a transformation process of array data was required to convert BGR into RGB. Then, the array was converted to 3D array (100 × 100 × 3) data to express the color values by horizontal and vertical pixels for each channel, and the dimension was reduced to the form of an array of 10,000 × 3 for easier analysis.

Clustering algorithms were applied to the main color analysis of sun object images. Clustering is an unsupervised learning technique that classifies similar data into groups even if information about input data is not given [33], and in the proposed method, clustering was performed by applying the K-means algorithm. The K-means algorithm has the advantage of being able to directly control the number of clusters, so it is suitable for detecting representative colors for sun object images. K-means algorithm was implemented through the sklearn library [34]. At this time, the number of clusters was set to five. Because the extracted size of the sun object image was twice the size of the detected sun in the horizontal and vertical directions, the final size of the sun region except for some background parts was about 20% of the size of the sun object image. When the number of clusters was set to five, the size of the priority cluster should be at least 20% or greater, to make the size of the representative color cluster equal to or larger than the size of the sun object. In this setting, there were cases where the size of clusters may be larger than that of the sun object, e.g., when the colors of clouds and sky were selected as the representative colors or when a sunset sky was inputted where the color of the sun was spread to the background. In fact, because the intensity of UV radiation reaching the Earth’s surface was related to the solar incidence angle and weather conditions around the sun, the setting was made to detect background elements in addition to the sun that affected UV radiation together in the representative colors.

### 2.3. DNN Model for UVI Calculation

A DNN model was developed to calculate the UVI by inputting RGB color values, which were the representative color of the sun object images derived from the previous section. For this purpose, additional input elements, which are highly related to the UVI, were selected and datasets for the learning and validation of the DNN model were built. As the components of the dataset, first, R, G, and B color values were selected, which were the representative colors of the changing sun object images, and the UVI was selected, which were measured together through a spectroradiometer at the time of acquisition of sun object images.

In addition, the UVI is closely related to the altitude and azimuth angles of the sun, and the intensity of hourly UV radiation reaching the Earth’s surface varies by month [35]. The information about the altitude and azimuth angles of the sun and month was selected as additional input elements. At this time, the altitude and azimuth angles of the sun were based on the measurement location and time of the sky images, and the values provided by the Korea Astronomical Research Institute were acquired and applied to the datasets. Table 2 presents some of the datasets that were built by accumulating and collecting major characteristics for the development of the DNN model that calculates the UVI.

The dataset in Table 2 consists of about 76,000 records after excluding the data collected before sunrise and after sunset, when the UVI converges to zero, from about 98,000 total collected images. Here, R, G, and B values and altitude and azimuth angles were difficult to be applied to the DNN model because their units were different. Thus, MinMaxScaling was applied to improve the learning speed and to reduce the overfitting probabilities. In addition, the One-Hot Encoding method was applied to input the form of categorical data for information about months. After that, the dataset was divided by a ratio of 4.9:2.1:3 for the learning, validation, and performance evaluation of the proposed model, respectively [36].

The deep learning model for UVI calculation was built through Keras, which employed TensorFlow as a backend. The deep learning model was a sequential structure that could be composed of a total of N layers including one input and one output layer. The number of nodes and connection structure in each layer containing hidden layers could be differently composed according to the number and format of data [37]. Before the implementation of the DNN model for UVI calculation, a pre-DNN model was implemented with various combinations of each input element, and the optimal input elements were derived through performance comparisons. The input layer of the pre-DNN model was implemented to accommodate up to 17 inputs in consideration of applying monthly (1–12) information using the One-Hot Encoding method. In addition, the number of hidden layers was set to two, which was the minimum number for implementing deep learning models, and the number of nodes in each layer was set to 16, which was the closest multiple of 2 to the number of input elements [38].

All layers of the pre-DNN model were composed of the dense layer, which presumed pre-combination, and ReLU was used for the activation function. The weight was initialized via uniform distribution and adam was adopted for the optimization algorithm. In the learning process, the loss function was set to mean absolute error (MAE), which was used as an evaluation criterion for comparing the performance of the pre-DNN model. The results of pre-experiments to find the optimal combination of input variables in the deep learning model for UVI calculation are presented in Table 3.

**Figure 3:**
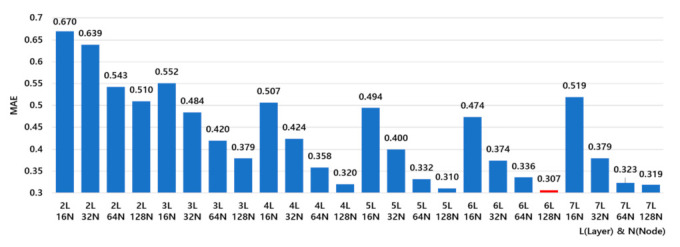
Grid search for model optimization.

In the pre-experiment results of Table 3, MAE was relatively lower at 1.52 in the 4th experiment, where three color elements were inputted together compared to those in the 1st–3rd experiments, where each of the color elements R, G, and B was independently inputted. In addition, when the values of the altitude angle and the zenith angle of the sun were applied as additional input elements, MAE was 0.74 and 1.12, respectively, and MAE was 0.9 when altitude and zenith angles were applied together. MAE was 0.53 when the month information was applied in addition, which showed the best performance. R, G, B, altitude, azimuth, and month were selected as the input variables of the proposed model, based on the results of the pre-experiments. In order to improve the performance of the proposed model, hyperparameter tuning was performed to optimize the number of hidden layers and the number of nodes. The hyperparameter tuning was performed using Grid Search functions of the sklearn library, which compares the performance of several deep learning models that combine properties within a predefined range [35]. For optimization, the number of nodes in the layer was set to 16, 32, 64, and 128, and the number of hidden layers was set to a range of 2 to 7. After that, Grid Search was performed to check the performance of the model by adjusting the number of hidden layers and nodes, and the results are shown in Figure 3.

**Figure 4:**
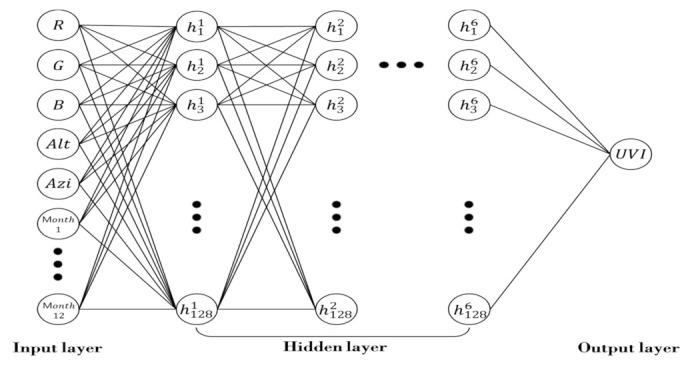
Structure of DNN models for UVI calculation.

In Figure 3, the *X*-axis refers to a model implemented by combining the number of layers (L) and the number of nodes (N) in each layer. On the *Y*-axis, MAE is expressed as an absolute value, which is the result of Grid Search in each layer and node condition. The more closely the absolute value of the MAE approaches zero, the better the performance of the model is. The proposed model showed the best performance when the number of hidden layers was six and the number of nodes was 128 (6L 128N), as the MAE was around 0.30. Then, the DNN model of Figure 4 was developed to calculate the UVI by inputting R, G, B, which were the representative colors of the sun object image, and altitude angle, azimuth angle, and monthly data, reflecting the results of Grid Search.

## 3. Experiments and Discussion

**Figure 5:**
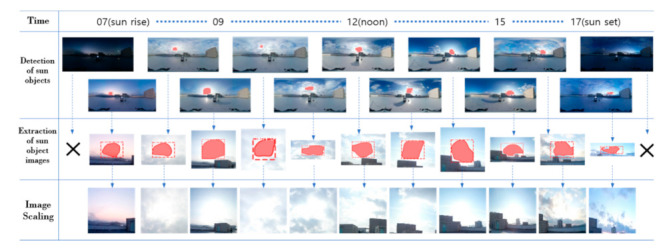
Detection and image scaling of sun object images for sky images over time (12 January 2020).

In the experiment, whether the sun object image and representative colors were extracted from sky images and the applicability of the deep learning model for UVI calculation were verified. First, the extraction performance of sun object images from a total of 98,000 sky images acquired during the data collection process was verified. Figure 5 shows an example of the result of performing the extraction and image scaling of sun object images from sky images photographed over one day.

Overall, there were many changes in clouds and weather, and sky images were also changed in various ways, such as sunset or overall darkening of the sky around sunrise (07:00) and sunset (19:00). Sun object images could be extracted from sky images at all times, except for the time when the sun was relatively dark before sunrise and after sunset. The extraction of sun object images was not possible from about 20,000 sky images including sunrise and sunset among all-sky images, but the UVI was found to be almost zero in those times; hence, the need to provide UV radiation information and the need to construct datasets were perceived to be low. In addition, it was verified that normalized images could be extracted after accurately detecting most of the sun object regions from a total of 70,000 sky images during the normal daytime, when UV radiation information is considered to be needed. The representative colors were extracted by applying K-Means clustering to about 70,000 previously extracted sun object images, and Table 4 shows some of the results.

**Figure 6:**
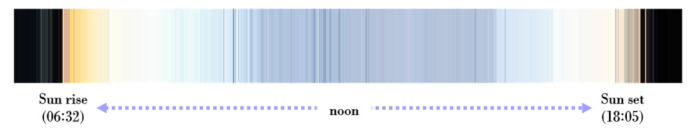
Detection results of major color components for sun object images over time (8 October 2020).

Table 4 shows that the sun object image near sunset, such as that shown in Table 4a, was detected with the main color components generated by sunset, and the main components of bright sky colors were mainly detected around noon, such as that shown in Table 4b,c. On cloudy days, as shown in Table 4d,e, gray-based main color components were detected. In the case of Table 4f, the sun image between sunrise and noon showed a relatively bright color similar to white. Different color clusters were formed in sun object images in various time and weather conditions, as shown in Table 4, and different RGB color values could be extracted as representative colors. The entire representative color extraction performance was shown for inputted sun object images. Figure 6 shows the extraction results of representative colors on a clear day (8 October 2020) which were expressed in spectral form by sequentially listing them by time. From sunrise to sunset, the main color of the sun object image appeared periodically in dark, bright, and sky blue, which seemed also highly related to the periodic changing intensity of UV radiation.

Experiments were conducted to check the UVI calculation performance of the proposed model, which applies representative colors of sun object images to the main input. In the case of Korea, the UV radiation is high in the order of summer, spring, autumn, and winter. In summer, when the levels of UV radiation are high, there is a seasonal characteristic that the atmosphere is unstable or rainy due to the influence of the rainy season. In summer, the proportion of datasets is relatively low because it is difficult to acquire sky images and UVIs. Winter also presents unfavorable conditions for acquiring experimental data due to snow and cloudy weather. In Korea, spring and autumn are recommended as good seasons for outdoor activities and there is a growing interest in UV radiation.

**Figure 7:**
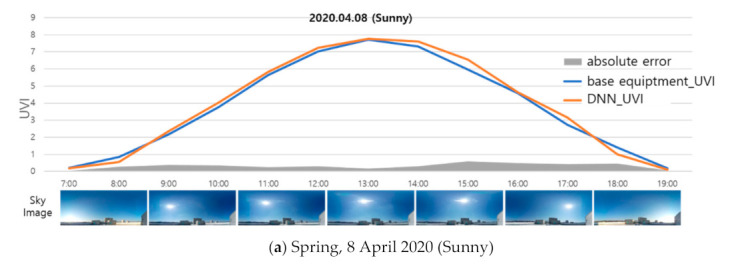

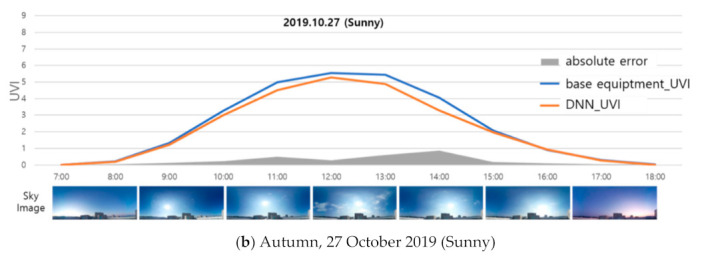
Performance evaluation of DNN model.

Considering these points, a clear day for spring and autumn was selected as the basic date for the experiment. The proposed method was applied to sky images acquired on the selected experiment day, and UVI at the same place and time was measured by a spectroradiometer (CAS 140CT). Figure 7 shows the result of comparing the results of applying the proposed model with the actual measurement result of UVI. For a fair performance evaluation of the proposed model, sun object images and UVIs acquired on that day were not applied to the learning of the model.

Figure 7a is the experiment result for a day of the spring season (8 April 2020). The UV index of the day measured by a spectroradiometer ranged from 0.0 at sunrise and sunset to 7.72 at noon. A total of 696 sky images were obtained on the same date, showing a clear overall day image pattern. The results of the proposed model, which calculated UVI values of 00.00~7.76, are very similar to the results of actual measurement by a spectroradiometer (0.0–7.72). The performance evaluation for the day (27 October 2019) in autumn showed that the results of the actual measurement for UVI are 0.0–5.53. The results of applying the proposed method to 637 sky images on the day show that the UV index was 0.0–5.28, and the results of actual measurement for UV index show similar values over time. The mean absolute error (MAE) for each experiment was 0.0–0.6 in spring, showing an average of 0.27, and was 0.0–0.9 in autumn, showing an average of 0.34.

According to the WHO guidelines, UVI is specified to be rounded from the first decimal digit and provided in integer units, and the Korea Meteorological Administration also applies the WHO guidelines. The proposed method can provide the information service of a UV index, which is the integer unit of the existing information service level for UV radiation. In addition, the average error of UVI was confirmed to be 0.3, which can provide a more detailed level of UVI information than existing information services for UV radiation. However, the Korea Meteorological Administration (KMA) operates seven UV-measuring stations across the country and provides the maximum UV index (average value) at 10 min intervals, so it is impossible to compare previous values with the present study’s results, which measure UV index at 1 min intervals (Figure 7). Although currently only UV information is provided at a specific location, it is possible to develop services through the regression-based correction of UV calculation results in other regions in the future.

## 4. Conclusions

Because UV radiation can benefit or harm human health depending on the degree of exposure, it is an environmental factor that must be checked during outdoor activities. However, as UV radiation is a non-visible wavelength band, unlike environmental factors such as weather and fine dust that can be determined by sight, it cannot be distinguished by the naked eye. It is very difficult for ordinary people to acquire UV information in their current position without using special UV radiometers. Herein, a DNN-based UVI calculation method was proposed using representative color information of sun object images. First, data of sky images and UVIs were collected at the same time and same place to analyze the correlation between UV radiation and images accurately. An omnidirectional camera (Gear 360, Samsung, Seoul, South Korea, and VIRB360, Garmin, Olathe, KS, USA) was applied for acquiring sky images. In addition, irradiance by wavelength was measured using a spectroradiometer (CAS 140 CT, Instrument Systems, Munich, Germany) and UVI was calculated by applying a function for erythema weight. After that, the sun object was detected by applying Mask R-CNN to the acquired sky images, and sun object images were extracted from a region corresponding to twice the size of a sun object and scaled to a size of 100 × 100 px. Then, the K-means clustering technique was applied to extract representative colors. At this time, colors in sun object images were classified into five clusters and the RGB values of the largest cluster were selected as the representative color. Then, a deep learning model was developed to calculate the UVI using the representative colors of sun object images. In addition to representative colors of sun object images, the position information of the sun and the categorical information about months were selected as additional input elements, and the DNN model was built to calculate the UVI by inputting the representative colors of sun object images, R, G, B, and the altitude and azimuth angles of the sun and month information. Then, the optimization of the DNN model consisting of 6 layers and 128 nodes in each layer was conducted through hyperparameter tuning. For performance evaluation, the results of applying the proposed model and measurement results of UVI collected by spectroradiometer were compared. As a result, in the comparison experiment for each clear day in spring and autumn, the mean absolute error (MAE) was 0.27 and 0.34, respectively, and accurate UVI information was calculated within an error range of less than 0.5 on average. In addition, the proposed model can provide outdoor UV radiation information with integer units or more detailed levels according to the WHO guidelines.

In the future, the measurement results of sky images and UVI for summer and winter, which were relatively difficult to construct datasets from, will be collected and applied to the proposed model to improve the performance of the deep learning model for UVI calculation. In addition, there is a limit to obtaining UV radiation information of the sun using panoramic images of cameras, which are now capable of omnidirectional measurement. For future studies, research will be conducted to generalize the proposed method so that information on UV radiation can be calculated even for smartphone-based acquired images.

## Figures and Tables

**Table 1:** Sky images and UVI by time.

Time	5 April 2020 08:00	5 April 2020 09:00	5 April 2020 10:00	5 April 2020 11:00	5 April 2020 12:00
Sky Image	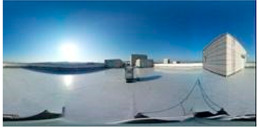	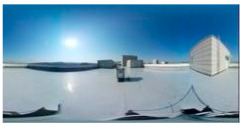	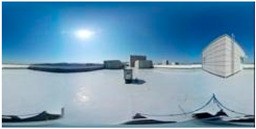	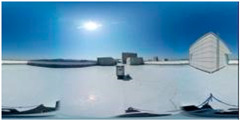	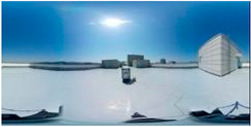
UVI	1.32316	2.83357	4.60154	6.26907	7.45116
**Time**	**5 April 2020 13:00**	**5 April 2020 14:00**	**5 April 2020 15:00**	**5 April 2020 16:00**	**5 April 2020 17:00**
Sky Image	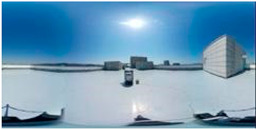	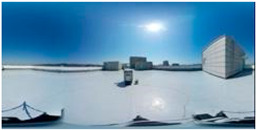	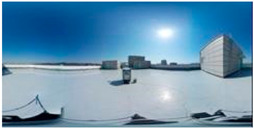	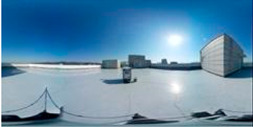	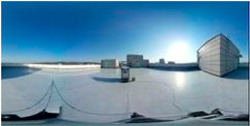
UVI	7.17109	6.84514	5.43987	3.73438	2.10725

**Table 2:** Dataset for building the DNN model for UVI calculation.

No.	R	G	B	Altitude	Azimuth	Month	UVI
1	0.99215	0.99607	0.98823	0.35106	0.30414	[1, 0, 0, 0, 0, 0, 0, 0, 0, 0, 0, 0]	2.73878
2	0.64705	0.71764	0.78039	0.29084	−0.27222	[1, 0, 0, 0, 0, 0, 0, 0, 0, 0, 0, 0]	1.15636
N-1	0.98823	0.98039	0.93725	0.12618	−0.41437	[0, 0, 0, 0, 0, 0, 0, 0, 0, 0, 0, 1]	0.70520
N	0.80784	0.85882	0.94509	0.51351	−0.10385	[0, 0, 0, 0, 0, 0, 0, 0, 0, 0, 0, 1]	7.25948

**Table 3:** Pre-experiments for optimal combination of input variables.

No.	R	G	B	Altitude	Azimuth	Month	MAE
1	O	X	X	X	X	X	1.97
2	X	O	X	X	X	X	1.96
3	X	X	O	X	X	X	1.68
4	O	O	O	X	X	X	1.52
5	O	O	O	O	X	X	0.74
6	O	O	O	X	O	X	1.12
7	O	O	O	O	O	X	0.59
8	O	O	O	O	O	O	0.53

**Table 4:** Major color analysis results for various sun object image types.

	(a)	(b)	(c)	(d)	(e)	(f)
Pre-processing Image	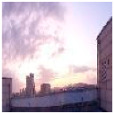	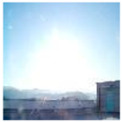	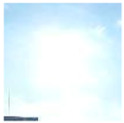	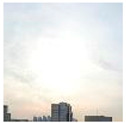	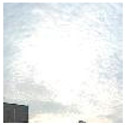	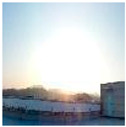
K-Means Clustering	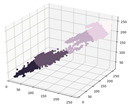	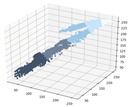	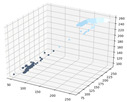	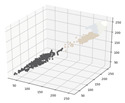	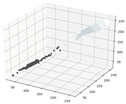	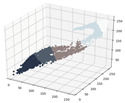
Result	116, 116, 167	142, 183, 216	30, 78, 145	165, 183, 201	144, 163, 183	246, 249, 248

## References

[1] Van der Rhee H., de Vries E., Coebergh J. (2016). Regular sun exposure benefits health. Med. Hypotheses.

[2] Lee K.-K., Park Y., Han S.-P., Kim H.C. (2020). The Alerting Effect from Rising Public Awareness of Air Quality on the Outdoor Activities of Megacity Residents. Sustainability.

[3] IARC Working Group (2006). Exposure to Artificial UV Radiation and Skin Cancer.

[4] Diffey B.L. (2002). Sources and measurement of ultraviolet radiation. Methods.

[5] World Health Organization, World Meteorological Organization, United Nations Environment Programme, International Commission on Non-Ionizing Radiation Protection (2002). Global Solar UV Index: A Practical Guide.

[6] Kim H.-S., Oh S.-T., Lim J.-H. (2018). Development of local area alert system against particulate matters and ultraviolet rays based on open IoT platform with P2P. Peer-to-Peer Netw. Appl..

[7] Tian J., He Y., Li J., Wei J., Li G., Guo J. (2018). Fast, Real-Time, In Situ Monitoring of Solar Ultraviolet Radiation Using Sun-light-Driven Photoresponsive Liquid Crystals. Adv. Opt. Mater..

[8] Kwak M.K., Kim J.H. (2011). The radiative characteristics of EUV-B over the Korean peninsula and exposure time for synthesizing adequate vitamin D. Atmosphere.

[9] Hooke R., Pearson A., O’Hagan J. (2014). Autonomous Portable Solar Ultraviolet Spectroradiometer (APSUS)—A New CCD Spec-trometer System for Localized, Real-Time Solar Ultraviolet (280–400 nm) Radiation Measurement. Photochem. Photobiol..

[10] Fahrni T., Kuhn M., Sommer P., Wattenhofer R., Welten S. Sundroid: Solar radiation awareness with smartphones. Proceedings of the 13th International Conference on Ubiquitous Computing.

[11] Park D.-H., Oh S.-T., Lim J.-H. (2019). Development of a UV Index Sensor-Based Portable Measurement Device with the EUVB Ratio of Natural Light. Sensors.

[12] Asare K.O., Leikanger T., Schuss S., Klakegg A., Visuri A., Ferreira D. S3: Environmental finger-printing with a credit card-sized NFC powered sensor board. Proceedings of the 20th International Conference on Human-Computer Interaction with Mobile Devices and Services Adjunct.

[13] Sosko S., Dalyot S. (2017). Crowdsourcing User-Generated Mobile Sensor Weather Data for Densifying Static Geosensor Networks. ISPRS Int. J. Geo-Inf..

[14] Igoe D.P., Parisi A.V., Amar A., Downs N.J., Turner J. (2018). Atmospheric total ozone column evaluation with a smartphone image sensor. Int. J. Remote Sens..

[15] Marquez R., Coimbra C.F. (2013). Intra-hour DNI forecasting based on cloud tracking image analysis. Sol. Energy.

[16] Liu X., Song Z., Ngai E., Ma J., Wang W., Xiaoyang L. PM2:5 monitoring using images from smartphones in participatory sensing. Proceedings of the 2015 IEEE Conference on Computer Communications Workshops (INFOCOM WKSHPS).

[17] Roshani S., Jamshidi M.B., Mohebi F., Roshani S. (2021). Design and Modeling of a Compact Power Divider with Squared Resonators Using Artificial Intelligence. Wirel. Pers. Commun..

[18] Roshani M., Sattari M.A., Ali P.J.M., Roshani G.H., Nazemi B., Corniani E., Nazemi E. (2020). Application of GMDH neural network technique to improve measuring precision of a simplified photon attenuation based two-phase flowmeter. Flow Meas. Instrum..

[19] Salman A.G., Kanigoro B., Heryadi Y. Weather forecasting using deep learning techniques. Proceedings of the 2015 International Conference on Advanced Computer Science and Information Systems (ICACSIS).

[20] Tao Y., Chen H., Qiu C. Wind power prediction and pattern feature based on deep learning method. Proceedings of the 2014 IEEE PES Asia-Pacific Power and Energy Engineering Conference (APPEEC).

[21] Jo Y.-S., Choi Y.-J., Kim M.-G., Woo C.-H., Min K.-W., Seon K.-I. (2021). Construction of a far-ultraviolet all-sky map from an in-complete survey: Application of a deep learning algorithm. Mon. Not. R. Astron. Soc..

[22] McKenzie R., Blumthaler M., Diaz S., Fioletov V., Herman J., Seckmeyer G., Smedley A., Webb A. (2014). Rationalizing Nomenclature for UV Doses and Effects on Humans.

[23] Gidaris S., Komodakis N. Object Detection via a Multi-region and Semantic Segmentation-Aware CNN Model. Proceedings of the 2015 IEEE International Conference on Computer Vision (ICCV).

[24] Girshick R., Donahue J., Darrell T., Malik J. Rich Feature Hierarchies for Accurate Object Detection and Semantic Segmentation. Proceedings of the IEEE Conference on Computer Vision and Pattern Recognition.

[25] He K., Gkioxari G., Dollár P., Girshick R. Mask r-cnn. Proceedings of the IEEE International Conference on Computer Vision.

[26] Matterport (2020). matterport/Mask_RCNN.10. https://github.com/matterport/Mask_RCNN.

[27] Kaiser L., Gomez A.N., Shazeer N., Vaswani A., Parmar N., Jones L., Uszkoreit J. (2017). One model to learn them all. arXiv.

[28] Lin T.Y., Maire M., Belongie S., Hays J., Perona P., Ramanan D., Dollár P., Zitnick C.L. Microsoft coco: Common objects in context. Proceedings of the European Conference on Computer Vision.

[29] Dutta A., Zisserman A. The VIA Annotation Software for Images, Audio and Video. Proceedings of the 27th ACM Interna-tional Conference on Multimedia.

[30] Ciresan D., Meier U., Schmidhuber J. Multi-column deep neural networks for image classification. Proceedings of the 2012 IEEE Conference on Computer Vision and Pattern Recognition.

[31] Szegedy C., Toshev A., Erhan D. Deep neural networks for object detection. Proceedings of the 26th International Conference on Neural Information Processing Systems.

[32] Krizhevsky A., Sutskever I., Hinton G.E. (2012). Imagenet classification with deep convolutional neural networks. Adv. Neural Inf. Process. Syst..

[33] Caron M., Bojanowski P., Joulin A., Douze M. Deep Clustering for Unsupervised Learning of Visual Features. Proceedings of the European Conference on Computer Vision (ECCV).

[34] Pedregosa F., Varoquaux G., Gramfort A., Michel V., Thirion V., Grisel O., Blondel M., Prettenhofer P., Weiss R., Dubourg V. (2011). Scikit-learn: Machine learning in Python. J. Mach. Learn. Res..

[35] Oh S.-T., Ga D.-H., Lim J.-H. (2021). Mobile Deep Learning System that Calculates UVI Using Illuminance Value of User’s Location. Sensors.

[36] Khagi B., Kwon G.R., Lama R. (2019). Comparative analysis of Alzheimer’s disease classification by CDR level using CNN, feature selection, and machine-learning techniques. Int. J. Imaging Syst. Technol..

[37] Wanas N., Auda G., Kamel M.S., Karray F. On the optimal number of hidden nodes in a neural network. Proceedings of the IEEE Canadian Conference on Electrical and Computer Engineering.

[38] Stathakis D. (2009). How many hidden layers and nodes?. Int. J. Remote Sens..

